# Coffin–Siris syndrome: Clinical description of two cases

**DOI:** 10.1002/ccr3.6598

**Published:** 2022-12-15

**Authors:** Nina Hollander, Gavin William ten Tusscher

**Affiliations:** ^1^ Department of Pediatrics and Neonatology Dijklander Hospital Hoorn The Netherlands; ^2^ Department of General Practice, Public Health and Methodology Amsterdam University Medical Center Amsterdam The Netherlands

**Keywords:** ARID1A gene, brachydactyly, Coffin–Siris syndrome, congenital birth defects, genetic testing

## Abstract

Coffin–Siris syndrome is a rare disorder, which can be difficult to recognize. A broad spectrum of nonspecific clinical features is associated with Coffin–Siris syndrome, and the expression of these features is diverse. We describe two cases with Coffin–Siris syndrome with mutations in the ARID1A gene, with dissimilar presentation and clinical course.

## Graphical abstract

This case report provides helpful insights into a rare syndrome. Providing physicians with these insights may contribute to earlier recognition of the syndrome.

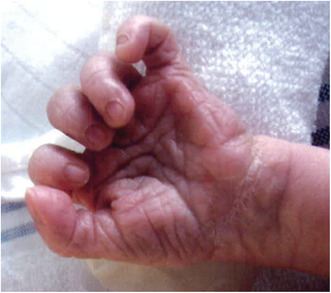

## 1 INTRODUCTION

Coffin–Siris syndrome is a rare genetic disorder of which the exact prevalence is unknown. Fewer than 250 cases of molecularly confirmed Coffin–Siris syndrome have been reported so far.^1^, ^2^ The syndrome was first described in 1970, and mutations associated with the syndrome were identified for the first time in 2012.^3^ Genes encoding proteins in the BAF (BRG1‐associated factor) chromatin‐remodeling complex mutations have been recognized as genetic cause for this syndrome, the most common being de novo mutations in the ARID1B gene on chromosome 6 (37% of confirmed cases reported).^4^ A broad spectrum of clinical features is associated with Coffin–Siris syndrome, including dysmorphic features, mental retardation and cerebral disorders, organ malformations, feeding problems, and frequent infections. The expression of the associated clinical features can be very diverse.

We describe two cases with confirmed Coffin–Siris syndrome, and mutations in the ARID1A gene were found in both cases. Clinical presentation and steps leading to the diagnosis were very different, although the patients do show similarities now and in retrospect also as newborns. Both the similarities in symptoms and the differences between the cases illustrate how difficult it can be to recognize Coffin–Siris syndrome, in addition to it being a rare disorder.

## 2 CASE DESCRIPTIONS

### 2.1 Case 1

At birth, neonatal resuscitation consisting of airway support and chest compressions was necessitated. The patient's condition improved quickly; her 1‐minute and 5‐minute APGAR scores were 3 and 8, respectively. She showed persisting signs of respiratory distress and dysmorphic features and was therefore admitted at a local neonatal unit postpartum.

An inspiratory stridor was observed. She became respiratory insufficient, was intubated, and admitted to a neonatal intensive care unit. It was suspected she had severe laryngomalacia; however, a partial choana atresia was found. A supraglottoplasty was performed when she was 1 month old.

**FIGURE 1:**
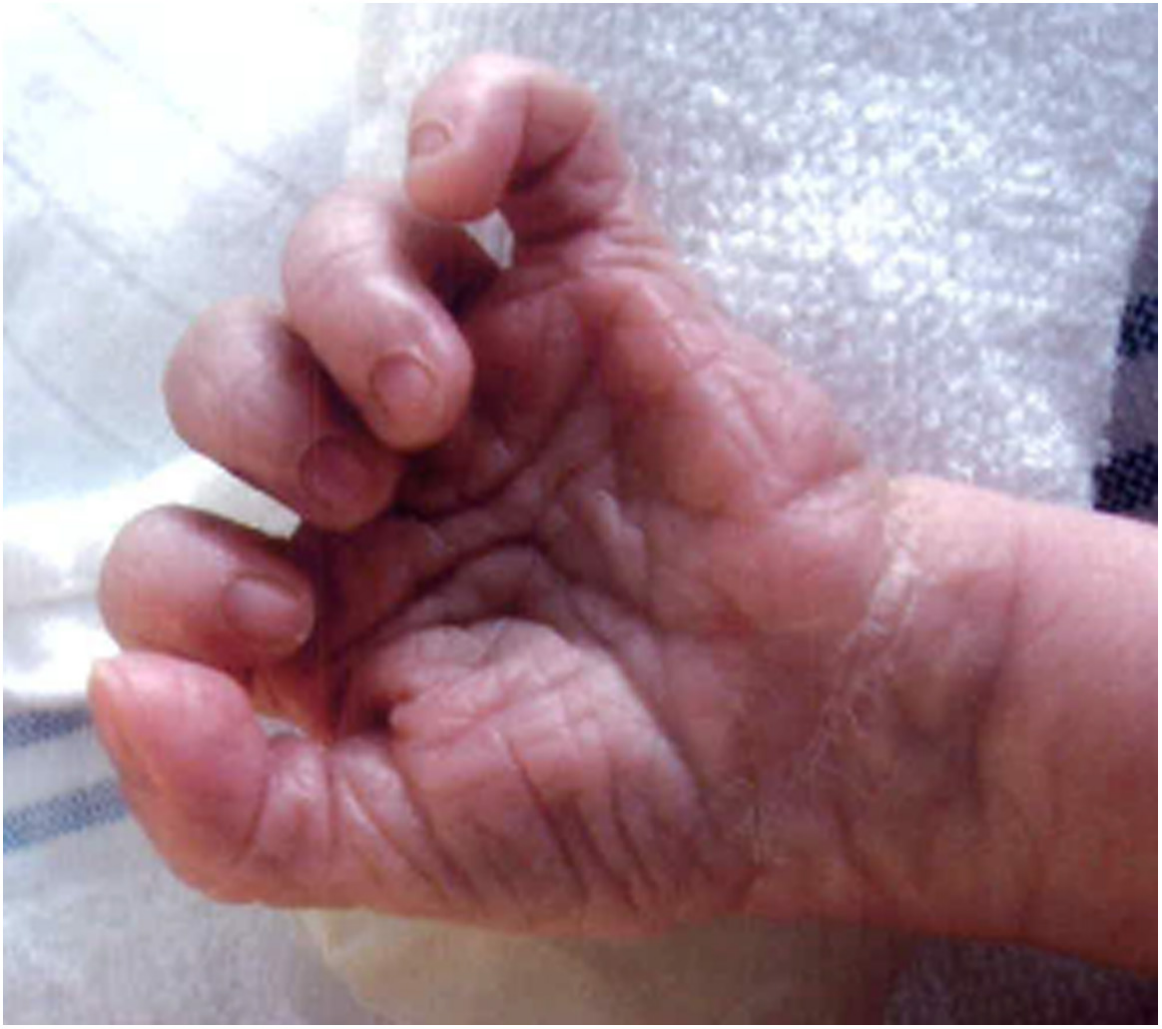
Clinodactyly and hypoplastic fingernail of the fifth digit

The dysmorphic features seen postpartum entailed a broadening of the skull and preauricular pits on both ears. Additionally, she had narrow fingers with clinodactyly and hypoplastic fingernails of the fifth digit on both hands (Figure 1). Furthermore, her toes showed slight irregularities. She was referred to a clinical geneticist. Performing chromosomal research, FISH, and micro‐array techniques, no abnormalities were found. No further diagnostics or follow‐up with the clinical geneticist were planned.

The patient showed developmental delays. She exhibited a delay in reaching motor skill milestones, including an inability to walk until she was 2 years and 2 months old. At the age of two and a half years, she was found to have agenesis of the corpus callosum. Special needs schooling was required due to mental retardation. Medical follow‐up was done mostly by a general practitioner and rehabilitation physician.

Throughout the first years of her life, she was frequently admitted to hospital, mainly due to infections. Infections generally had a complicated course, often resulting in severe electrolyte disturbances due to vomiting and refusal to swallow liquids and even saliva, compounded with Kussmaul's breathing. She was referred to and seen by numerous doctors, including pediatricians, a neurologist, cardiologists, ENT and opthalmologists, and other professionals during and after admissions.

In the search for an explanation for her developmental delay and mental retardation and the abnormal course of her illnesses, the patient was referred to a clinical geneticist for the second time when she was 7 years old. Additional genetic and metabolic tests were performed. Via whole‐exome sequencing, a mutation in the ARID1A gene was found. It was concluded that she has Coffin–Siris syndrome.

### 2.2 Case 2

This newborn girl required some assistance at birth, consisting of airway management, which could successfully be stopped after a few minutes. Her condition improved quickly; her 1‐minute and 5‐minute APGAR scores were 3 and 7, respectively. She was admitted for observation at the local neonatal unit. Besides “noisy” breathing, thought to be caused by nasal congestion, she showed no abnormalities. She was discharged from hospital after 2 days.

At the age of 2 weeks, the persistent “noisy” breathing was the reason for referral to an ENT surgeon. Laryngomalacia was diagnosed. No interventions were required as she was clinically in good condition.

At the age of 2 months, the patient was admitted to hospital with a failure to thrive. She had been breastfed. Her growth was insufficient, and she increasingly showed signs of tiredness and quickly fell asleep during feeding. It was suspected that the failure to thrive was due to inadequate intake and an increased energy need as a result of her laryngomalacia. Various interventions for increasing her caloric intake were undertaken. She was transferred to an academic medical center. A clinical geneticist performed chromosomal analysis and single nucleotide polymorphism (SNP) arrays. The patient's parents were consanguine. The patient was discharged with feeding via a nasogastric tube. Her weight gain had normalized.

The patient was frequently admitted to hospital in the following months, mainly due to infections and gastrointestinal problems. Two months after the previously described hospital discharge, she was admitted due to cyanotic incidents. The most probable diagnosis was laryngeal spasms related to both her laryngomalacia and gastroesophageal reflux. She was observed and prescribed omeprazole and erythromycine. At the age of 10 months, she was again admitted and transferred to an academic medical center; this time due to juvenile idiopathic arthritis. She was started on anakinra and responded well to the treatment.

The clinical genetics tests revealed no abnormalities. However, subtle dysmorphic features became more evident: brachycephaly, uplifted earlobes, thick eyebrows and long eyelashes, fetal pads on the fingers and deep palmary lines, hypopigmentations, and small toenails of the fifth digit. Metabolic diagnostics and whole‐exome sequencing were performed. At the age of one and half years, the diagnosis of Coffin–Siris syndrome was made. A mutation in the ARID1A gene was found through whole‐exome sequencing.

## 3 DISCUSSION

### 3.1 Importance of top‐to‐toe physical examination

Similar in both cases is the fact that doctors, including clinical geneticists, were unable to diagnose the patients without genetic testing. The cases illustrate, as is known in Coffin–Siris syndrome, that dysmorphic clinical features in patients may be subtle, diverse, and not specific enough for easy diagnosis (Table 1). A number of dysmorphic features were prominent in Case 1, yet they were not specific enough to diagnose her as having Coffin–Siris syndrome. In Case 2, the dysmorphologies were not recognized by physicians who had seen her prior to referral to a clinical geneticist, who also found insufficient evidence to diagnose her initially. Besides dysmorphologies that could have been found in infancy, the patients showed signs in early childhood that increased the likelihood of them having a genetic disorder. Both patients showed developmental delay and intellectual disability, both unspecific features of Coffin–Siris syndrome. The patients were frequently admitted to hospital due to infections and gastrointestinal problems, which were often with more severe illness than in average children. In Coffin–Siris syndrome specifically, feeding problems are described to occur in 90% of the cases in infancy and frequent infections in 60% of the cases.^4^ Both patients had laryngomalacia, which Coffin–Siris syndrome is associated with, but which is also seen frequently in many other syndromes or also as isolated abnormality. The patients were examined frequently and thoroughly by doctors, both because of the increased likelihood of them having a genetic syndrome and due to their infections. Despite that awareness, dysmorphologies were missed.

**TABLE 1:** Physical features associated with Coffin–Siris syndrome found in infancy and early childhood^4^

Findings in infancy	Findings in early childhood
Brachydactyly of the fifth digit/hypoplasia of ≥1 nail (80%)	Brachydactyly of the fifth digit/hypoplasia of ≥1 nail (80%)
Dysmorphic facial features (30%)	Dysmorphic facial features (≥95%)^a^
Hirsutism (unknown)	Hypertrichosis (95%), low anterior hairline (75%)
Hypotonia (75%)	Hypotonia (75%)
	Ophtalmologic abnormalities such as ptosis (50%) or strabismus (50%)
	Musculoskeletal abnormalities other than fifth digit abnormalities^b^

^a^ Dysmorphic facial features associated with Coffin–Siris syndrome include coarse facies (95%), thick eyebrows (90%), prominent eyelashes (85%), flat nasal bridge (50%), short nose (50%), anteverted nares (50%), broad nasal tip (75%), wide nasal base (50%), thick alae nasi (70%), broad philtrum (70%), wide mouth (80%), thin vermilion of the upper lip (50%), thick vermilion of the lower lip (80%).

^b^ Clinodactyly (40%), joint laxity (66%), scoliosis (30%).

This illustrates the value of genetic testing and thereby recognition of genetic causes for this syndrome, discussed further below. Simultaneously, the cases, or Coffin–Siris syndrome itself, highlight the importance of a thorough “top‐to‐toe” physical examination. Coffin–Siris syndrome is also known as the “fifth digit syndrome,” because it is associated with abnormalities of the fifth fingers and/or toes. Both our cases had hypoplastic fifth digits. In Case 1, this was recognized shortly after birth. Nonetheless, among the other dysmorphic features and symptoms, this was not sufficient to recognize the patient as having Coffin–Siris syndrome. In Case 2, the clinodactyly was not recognized until she was one and a half years old, when the clinical geneticist performed either a more complete or a more critical physical examination. As described by Vergano et al.,^4^ most patients with Coffin–Siris syndrome have a minimal brachydactyly of the fifth digit (seen in 65% of affected infants) and hypoplasia of one or more nails (80%). Although it is impossible to claim that the patients could have been diagnosed without whole‐exome sequencing, it is interesting to note how these subtle dysmorphic features were missed. As physicians, we are taught to perform top‐to‐toe physical examinations in newborns. In both cases, physical examination was frequently performed and reported, especially because of the multiple problems and suspected genetic cause, yet the fifth digit anomalies were not or insufficiently noticed. Clinodactyly and hypoplastic fingernails may be difficult to recognize in newborns because of the small size of their digits and nails. Especially, while searching for dysmorphic features in patients with multiple morbidities or patients already suspected of having a syndrome, it is therefore important to look critically from top to every toe.

### 3.2 Prevalence/recognition

As described in the introduction, the prevalence of Coffin–Siris syndrome is unknown. This can be both due to the rarity of the disorder, and it is insufficiently recognized in patients. Patients with Coffin–Siris syndrome may go unrecognized, as illustrated by Case 1 who was not diagnosed until she was 7 years old and might never have been diagnosed if she had not been referred to a clinical geneticist for a second time. These patients will often be diagnosed as patients with intellectual disability of unknown etiology, or patients with suspected genetic disorder of unknown etiology. For Coffin–Siris syndrome, as well as for other syndromes, etiological diagnosis is considered beneficial to establish. A number of benefits of establishing an etiologic diagnosis are described for patients with developmental delay and/or intellectual disability by Moeschler et al.,^5^ such as provision of prognosis, discussion of genetic mechanisms and recurrence risks, the avoidance of unnecessary and redundant diagnostic tests and acquiring information regarding symptom management, and surveillance for known complications.

In both cases, genetic counseling was a relevant benefit for the families after establishing the genetic cause. Coffin–Siris syndrome is caused by a heterozygous pathogenic variant in one of the recognized genes and is inherited in an autosomal dominant manner. It most commonly results from a de novo pathogenic variant. If the pathogenic variant is identified in a family member, prenatal testing for a pregnancy at increased risk and preimplantation genetic testing are possible.^4^ In Case 2, the patient was just one and a half years old when diagnosed, Case 1 was 7 years old, meaning this information could still help both families make decisions concerning possible future pregnancies. In Case 2, this information was considered extra relevant because the parents were consanguine, and risk of a genetic disorder was thereby expected to be increased.

Avoidance of unnecessary and redundant diagnostic tests is relevant in Coffin–Siris syndrome, best illustrated by Case 1. Case 1 was referred to numerous specialists of whom many performed, in hindsight, redundant diagnostic tests and could have taken a different approach to their management had they known her diagnosis.

Finally, adequate information regarding symptom management and surveillance for known complications, provided by a clinical geneticist, was beneficial in both cases.

Since genetic causes for the syndrome were identified for the first time in 2012, this may lead to more patients being diagnosed and patients being diagnosed earlier in life. In turn, it is possible this will lead to more information concerning the prevalence and adequate advice concerning the benefits of establishing the etiological diagnosis described above. We hope more information will help improve the care for our patients, as well as possibly future patients with Coffin–Siris syndrome.

## AUTHOR CONTRIBUTIONS

Nina Hollander, MD is primary author of the manuscript. Gavin William ten Tusscher, MD, PhD, pediatrician is the treating physician of both patients. He supervised the writing and editing of the manuscript. He is the corresponding author.

## CONFLICT OF INTEREST

No conflicts of interest.

## CONSENT

The parents of both patients gave written informed consent for the usage of the patient data.
